# Supplementary material for: Validation of the T Descriptor (TNM-8) in T3N0 Non-Small-Cell Lung Cancer Patients; a Bicentric Cohort Analysis with Arguments for Redefinition
Source: Cancers (Basel). 2021 Apr 10;13(8):1812. doi: 10.3390/cancers13081812 (PMC8068959; doi:10.3390/cancers13081812)
Supplement: Supplementary file 1 [file cancers-13-01812-s001.pdf]

cancers

MDPI

Supplementary materials: Validation of the T Descriptor (TNM-8) in T3N0 Non-Small-Cell Lung Cancer Patients; a Bi-centric Cohort Analysis with Arguments for Redefinition

Philip Baum, Samantha Taber, Stella Erdmann, Thomas Muley, Mark Kriegsmann, Petros Christopoulos, Michael Thomas, Hauke Winter, Joachim Pfannschmidt and Martin E. Eichhorn

Table S1. Differences between Patients with chemotherapy vs. no chemotherapy. Patients with adjuvant Radio-Chemotherapy/ Radiotherapy are not included in the analysis.

	No chemotherapy		chemotherapy		
	**No.**	%	**No.**	%	**p value**
**All**	**109**	40.7	**159**	59.3	
**Gender**					*0.940*
Male	**66**	60.6	**97**	61.0	
Female	**43**	39.5	**62**	39.0	
**ECOG**					*<0.001*
0	**75**	68.8	**147**	92.5	
>=1	**34**	30.3	**12**	7.6	
**Age**					*0.155*
<60	**8**	7.3	**24**	15.1	
60-69	**25**	22.9	**32**	20.1	
>=70	**76**	69.7	**103**	64.8	
**Surgery**					*0.749*
Lobectomy	**89**	81.7	**135**	84.9	
Sleeve Resection	**3**	2.8	**3**	1.9	
Sublobar Resection	**7**	6.4	**6**	3.8	
Bilobectomy	**3**	2.8	**7**	4.4	
Pneumonectomy	**7**	6.4	**8**	5.0	
**Resection side***					*0.310*
Right	**68**	62.4	**91**	57.2	
Left	**40**	36.7	**68**	42.8	
**Residue**					*0.190*
0	**105**	96.3	**157**	98.7	
1	**4**	3.7	**2**	1.3	
**Histology**					*0.586*
AdC	**49**	45.0	**77**	48.4	
SqCC	**47**	43.1	**59**	37.1	
Other	**13**	11.9	**23**	14.5	

*one Patient with missing side.
